# Active Components and Pharmacological Effects of *Cornus officinalis*: Literature Review

**DOI:** 10.3389/fphar.2021.633447

**Published:** 2021-04-12

**Authors:** Xue Gao, Yi Liu, Zhichao An, Jian Ni

**Affiliations:** ^1^ School of Chinese Materia Medica, Beijing University of Chinese Medicine, Beijing, China; ^2^ School of Chinese Materia Medica, Tianjin University of Chinese Medicine, Tianjin, China; ^3^ Dongzhimen Hospital, Beijing University of Chinese Medicine, Beijing, China

**Keywords:** *Cornus officinalis*, pharmacological effects, neurodegenerative diseases, diabetic nephropathy, traditional Chinese medicine

## Abstract

*Cornus officinalis* Sieb. et Zucc. (Shanzhuyu), a herb and food plant in east Asia, has the properties of tonifying the liver and kidney, and nourishing the essence according to the theory of traditional Chinese medicine. *C. officinalis* has been commonly used to treat asthenia diseases, liver, and kidney diseases, and reproductive system diseases since ancient times. The objectives of this article were to review the pharmacological effects and phytochemistry of *C. officinalis*. We conducted a literature review of the pharmacological effects of *C. officinalis* by different systems and compared the effects with the traditional usages, discussed the research status and potential blanks to be filled. The experimental studies showed that *C. officinalis* extract and its active components had various pharmacological effects such as anti-oxidation, anti-apoptosis, anti-inflammation, anti-diabetes, anti-osteoporosis, immunoregulation, neuroprotection, and cardiovascular protection, but clinical studies are still needed to assess whether the reported pharmacological activities have confirmed efficacy.

## Introduction

*Cornus officinalis* Sieb. et Zucc. (Shanzhuyu) is a herb and food plant used in traditional Chinese medicine (TCM). *C. officinalis* has a sour and astringent taste (Liu et al., 2011). In the theory of TCM, *C. officinalis* belongs to the liver and kidney meridians and has a mild warm nature. It is often used to tonify the liver and kidney, and arrest the loss of essence (Di et al., 2018). Because of its excellent tonic effect, *C. officinalis* has been commonly used to treat asthenia, liver, and kidney diseases, and reproductive system diseases since ancient times. Pharmacological studies have found that *C. officinalis* extract has a variety of biological activities such as anti-inflammatory, anti-oxidative, anti-apoptotic, anti-diabetic, neuroprotective, and cardiovascular protective activities. There are links and differences between these biological effects and the traditional usages of *C. officinalis*. (Czerwinska and Melzig, 2018) suggested that different cultural needs resulting from the different geographical distributions of the medicinal herbs could influence the research directions and results. Therefore, a literature review that combines the results of modern pharmacological research with the TCM theories has a certain value. The extract of *C. officinalis* is a collection of one or several groups of phytochemicals. Unlike single active components, the extract of herbal plant maintains the TCM characteristics of complex components and multiple targets (Liu et al., 2020a). The objectives of this article were to review the pharmacological effects of the extract and active components of *C. officinalis* separately according to published studies, and discuss the potential blanks to be filled.

Studies on *C. officinalis* published before December 2020 were gathered by searching the online databases (PubMed, Web of Science and Baidu Scholar) using the keyword “*Cornus officinalis*” and “Shanzhuyu” (Chinese pinyin of *C. officinalis*).

## Pharmacological Effects of *C. officinalis* Extract

### Diabetes

In TCM point of view, *C. officinalis* has the effects of restraining essence loss and reducing urination, and is often used together with *Radix Rehmanniae* (Dihuang). *C. officinalis* has been used to treat symptoms such as thirst, frequent urination, and “drink more, urinate more” since ancient times, and these symptoms nowadays are known as some typical symptoms of diabetes mellitus (DM) (Di et al., 2018). Pharmacological studies have found that *C. officinalis* extract has a significant hypoglycemic effect. (Park et al., 2011a) found that *C. officinalis* extract had an *α*-glucosidase inhibitory activity, and that it could improve oral sucrose tolerance and inhibit plasma glucose level increase in normal rats. (He, 2011) suggested that the hypoglycemic effect of *C. officinalis* was attributable to its total iridoid glycosides. They compared the total iridoid glycosides and the *α*-glucosidase inhibitory effects of crude and wine-processed *C. officinalis*. The results showed that the content of total iridoid glycosides of the crude *C. officinalis* was higher than that of the wine-processed *C. officinalis* (75.3 vs. 42.8%), and the *α*-glucosidase inhibitory effect of the crude *C. officinalis* was superior to that of the wine-processed *C. officinalis*, which confirmed their hypothesis.

*C. officinalis* extract has also been demonstrated to promote glucose uptake of cells. (Lau et al., 2008) found that aqueous extract of *C. officinalis* could stimulate glucose uptake of human skin fibroblasts Hs68 cells and mice adipose 3T3-L1 cells *in vitro*, but had no significant effects on glucose absorption of intestinal brush border membrane vesicles (BBMV). (Qian et al., 2001) found that ethanol extract of *C. officinalis* could increase the protein and mRNA expression of glucose transporter 4 (GLUT4) in skeletal muscle of DM rats induced by streptozotocin (STZ) and promote the transmembrane transport of glucose.

Gluconeogenesis is the major source of glucose output of the liver (Hatting et al., 2018). The effect of *C. officinalis* extract on hepatic gluconeogenesis remains controversial. Phosphoenolpyruvate carboxykinase (PEPCK) is a gluconeogenic rate-limiting enzyme. (Chen et al., 2008) found that phenolic compounds in the methanol extract of *C. officinalis* could up-regulate the gene expression of PEPCK and inhibit hepatic gluconeogenesis. However, the research of (Lau et al., 2008) suggested that *C. officinalis* extract had no significant effect on hepatic gluconeogenesis.

Insulin is the only hypoglycemic hormone in the human body, which can inhibit hepatic gluconeogenesis and glycogen decomposition, promote cell glucose uptake, and is important for maintaining normal glycolipid metabolism (Hatting et al., 2018). Insulin deficiency or insulin resistance is the direct cause of DM. In patients or model animals with type 2 diabetes, insulin resistance abnormally increases serum insulin levels (Jia et al., 2016). The research of (Liu et al., 2012a) showed that total saponins in *C. officinalis* extract reduced the blood glucose and insulin levels in STZ-induced diabetic rats. *C. officinalis* has been demonstrated to have islet *β*-cell protection effect, it can improve the pathological damage of the pancreas, increase the number of insulin releasing *β*-cells, and increase glucose-stimulated insulin secretion (Han et al., 2014). (Sharp-Tawfik et al., 2019) found that *C. officinalis* extract could inhibit cytokine-mediated *β*-cell death and improve *β*-cell function via up-regulating the expression of nuclear factor of activated T cells, cytoplasmic 2 (NFATC2).

As discussed above, the main components of *C. officinalis* that play a role in reducing blood glucose are iridoid glycosides, polyphenols, and saponins. *C. officinalis* extract reduces the absorption of glucose by small intestinal chorionic epithelial cells by inhibiting the activity of *α*-glucosidase; on the other hand, it promotes glucose uptake and utilization by promoting insulin secretion and improving insulin sensitivity of peripheral tissues, and inhibits gluconeogenesis.

In addition, *C. officinalis* extract can also decrease blood lipid levels, and it showed the effects of reducing the levels of total cholesterol (TC), triglycerides (TG), and low-density lipoprotein (LDL) of STZ-induced diabetic rats, and marginally increasing high-density lipoprotein (HDL) (Gao et al., 2012).

### Diabetic Complications

The combination of advanced glycosylation end products (AGEs) and AGE-receptor (RAGE) can cause the activation of endothelin (ET) system (Adamopoulos et al., 2016) and inducible nitric oxide synthase (iNOS) (Tang et al., 2016), and the accumulation of reactive oxygen species (ROS) (Koulis et al., 2015). This will impair vasodilation function and lead to microvascular and macrovascular complications. (Su et al., 2007) found that total triterpene acids isolated from *C. officinalis* could down-regulate mRNA expression of prepro-endothelin-1 (ppET-1), endothelin-converting enzyme (ECE) and iNOS in thoracic aorta and mRNA expression of ET_A_ receptor and iNOS in retina in diabetic rats. It increased acetyl choline (Ach)-mediated vasodilatation and nitric oxide (NO) bioavailability, and ultimately improved vasodilation function. (Liu et al., 2012b) found that total saponins of *C. officinalis* could decrease the contractile responsiveness to phenylephrine of the mesenteric artery rings isolated from STZ-induced diabetic rats, increase the diastolic reaction to Ach, and improve the baseline release of NO of the mesenteric artery.

It can be seen from the above that the triterpene acids and saponins from *C. officinalis* improve the vascular complications of DM mainly by regulating the vasodilation function. Since *C. officinalis* has specific kidney and liver protective effects, the therapeutic effect on diabetic nephropathy (DN) and DM liver injury are discussed separately in the following sections.

### Diabetic Nephropathy

The results of experimental studies on DN showed that AGEs/RAGE signaling pathway in diabetic complications could be regulated by *C. officinalis*. (Yamabe et al., 2007a; Yamabe et al., 2007b) found that the iridoid glycosides and low molecular weight polyphenols extracted form *C. officinalis* could reduce the urine protein and serum creatinine (Scr) levels of STZ-induced DM rats by decreasing the levels of AGEs, RAGE, nuclear factor kappa-B (NF-κB), and transforming growth factor-*β* (TGF-*β*) in the kidney tissue. The research of (Lv et al., 2016a) showed that the iridoid glycosides and triterpenoid acid in *C. officinalis* decreased the levels of 24 h urine protein, blood urea nitrogen (BUN), and Scr in db/db mice by down-regulating the activities of AGEs/RAGE/SphK1 pathway and TGF-*β*.

The research of (Qi et al., 2014) showed that although total triterpene acids of *C. officinalis* had no significant effect on blood glucose of DM rats, they could reduce urinary protein, Scr, and BUN levels, and improve renal pathological changes. They found that total triterpene acids of *C. officinalis* down-regulated malondialdehyde (MDA) level, up-regulated superoxide dismutase (SOD), catalase (CAT), and glutathione peroxide (GSH-px) activities; and decreased TGF-*β*1 expression in renal tissues of DM rats. The results indicated that total triterpene acids of *C. officinalis* could improve the diabetic kidney injury by antioxidant effect rather than hypoglycemic effect.

Peroxisome proliferator-activated receptor-γ (PPARγ) is a member of the nuclear hormone receptor superfamily of ligand-activated transcription factors and plays important roles in the regulation of glycolipid metabolism and insulin sensitivity. PPARγ agonist ligands can down-regulate the expression or activity of nicotinamide adenine dinucleotide (NAD) oxidase. PPARγ activators, such as rosiglitazone, are widely used in the treatment of type 2 DM (Ahsan, 2019). The research of (Gao et al., 2012) found that the ethanol extract of *C. officinalis* could elevate renal PPARγ expression in DM rats and improve the kidney injury caused by oxidative stress.

*C. officinalis* extract has protective effects on intrinsic renal cells. (Liu et al., 2012a) found that *C. officinalis* extract could increase the glomeruli expression of podocyte-specific marker Wilms tumor type 1 (WT1) in STZ-induced DM rats, indicating a capacity to protect the podocytes. The *in vitro* research of (Ma et al., 2014) showed that *C. officinalis* extract could inhibit the expressions of collagen IV (Col V), fibronectin (FN), and Interleukin-6 (IL-6) in high glucose induced HBZY-1 mesangial cells, revealing a protective effect of *C. officinalis* extract on the mesangial cells, and reducing the accumulation of mesangial matrix.

*C. officinalis* extract can also relieve DN renal fibrosis. The research of (Xu and Hao, 2004) showed that total iridoid glycosides extracted from *C. officinalis* could decrease serum TGF-*β*1 level and renal TGF-*β*1 mRNA expression and suppress the overdeposition of FN and laminin (LN). The research of (Han et al., 2014) showed that *C. officinalis* extract could inhibit the expression of fibroblast marker protein alpha-smooth muscle actin (*α*-SMA) in the kidney tissues of DN rats.

Our review of the literature showed that iridoid glycosides, low molecular weight polyphenols, and triterpene acids in *C. officinalis* had anti-inflammatory, anti-oxidative, anti-apoptotic and anti-fibrosis effects by regulating the AGEs/RAGE, NF-κB/TGF-*β*, and PPARγ signaling pathways, thereby alleviating the injury of the intrinsic renal cells.

### Liver Disease

In TCM point of view, sour taste enters the liver, so *C. officinalis* is often used to treat liver diseases. (Park et al., 2015a) found that *C. officinalis* could alleviate DM-induced liver damage. Their results showed that *C. officinalis* significantly decreased the elevated serum and hepatic glucose concentrations in diabetic rats, decreased the hepatic RAGE and AGEs expressions, reduced the abnormal accumulation of ROS and lipid peroxide, down-regulated the hepatic overexpression of nicotinamide adenine dinucleotide phosphatase oxidase 4 (NOX4), p22^*phox*^, nuclear factor erythroid-2 related factor 2 (Nrf2), heme oxygenase-1 (HO-1), NF-κB, cyclooxygenase-2 (COX-2), iNOS, monocyte chemoattractant protein-1 (MCP-1), and intercellular adhesion molecule-1 (ICAM-1), thereby inhibiting oxidative stress and inflammation responses.

*C. officinalis* has also been demonstrated to prevent acute liver injury. (Lee et al., 2012) pretreated BALB/c mice with the ethanol extract of *C. officinalis* orally for seven consecutive days before acute liver injury was induced in experimental mice by intraperitoneal (i.p) injection of 200 mg/kg acetaminophen (APAP). The results showed that *C. officinalis* extract reduced serum AST, ALT, LDH, and MDA levels, elevated the levels of antioxidant enzyme SOD, CAT, and GSH, indicating that *C. officinalis* improved APAP-induced acute liver injury by preventing or alleviating oxidative stress.

### Diseases of the Nervous System

According to the theory of TCM, the brain is born from the essence of the kidney. Therefore, the deficiency of kidney essence will lead to insufficiency of the brain and the symptoms of amnesia, dementia, and hemiplegia, which are the manifestations of central nervous system diseases in modern medicine. Experimental studies have confirmed that *C. officinalis* has therapeutic effects on neurodegenerative diseases such as Alzheimer's disease (AD) and depression. Glutamate can cause neuronal cell death and neurodegenerative diseases through ROS-mediated oxidative glutamate toxicity (Cassano et al., 2016). The study of (Jeong et al., 2012) indicated that the iridoid glycosides of *C. officinalis* had a significant protective effect against glutamate-induced toxicity in the hippocampal cells. The death of the cholinergic neurons in the basal forebrain area is considered to be one of the main causes of cognitive disorders, including AD. The study of (Lee et al., 2009) found that the methanol extract of *C. officinalis* and its main component, loganin, alleviated the memory loss in mice with amnesia induced by scopolamine, an anticholinergic drug. (Bhakta et al., 2017) found that several compounds isolated from *C. officinalis* could interact with both the catalytic active sites and the peripheral anionic sites of cholinesterase (ChEs) and the beta-site of amyloid precursor protein cleaving enzyme 1 (BACE1), leading to the inhibition of the activities of ChEs and BACE1.

So far, two major pathological lesions have been found in the brains of AD patients, amyloid plaques composed of amyloid-*β* (A*β*) peptides, and neurofibrillary tangles (NFTs) caused by hyperphosphorylated tau protein. Tau is an important structural protein of neurons, tauopathies are well known to impair the transmission of nerve impulses (Naseri et al., 2019). The study of (Yang et al., 2020) used the APP/PS1/tau triple transgenic(3 × Tg) mice as the rodent model of AD, and they found that the iridoid glycoside extracted from *C. officinalis* could ameliorate learning and memory impairment of 3 × Tg mice by down-regulating the expressions of A*β* and full-length amyloid precursor protein, as well as decreasing the hyperphosphorylation of tau protein. (Ma et al., 2020) got the similar results that the iridoid glycoside of *C. officinalis* could reduce the hyperphosphorylation and aggregation of tau protein in amygdala and prevent neuronal loss, and suppressed hyperactivity phenotype of AD.

PC12 cells, derived from the pheochromocytoma of the adrenal medulla in rats, have typical neuron characteristics and are widely used in studies of neurological diseases. Corticosterone-induced PC12 injury is considered an *in vitro* experimental model for depression (Tian et al., 2018). The study of (Ji et al., 2019) indicated that iridoid glycosides of *C. officinalis* could reduce corticosterone-induced PC12 cell damage *in vitro*. Oxidative stress plays an important role in neurodegenerative diseases, and stress often lead to excessive accumulation of ROS. Immobilization stress is widely used to induce depressive-like and anxiety-like behaviors and hippocampal neuronal damage in rodents (Seo et al., 2012). (Tian et al., 2019) utilized immobilization stress to investigate the protective effect of *C. officinalis* extract on nerve injury, and the results showed that *C. officinalis* effectively alleviated the oxidative stress, decreased immobility time in forced swim test (FST), and significantly reduced the levels of corticosterone and β-endorphin, and increased the level of serotonin, which indicated that apart from regulating the cholinergic signaling pathway, *C. officinalis* could also treat neurodegenerative diseases through antioxidant effects.

In addition, *C. officinalis* can also reduce the damage caused by cerebral infarction. (Li et al., 2005) found that the area of infarction was reduced in rats fed with *C. officinalis* extract for 7 days before cerebral infarction was induced, and the effect was related to the decrease in NOS and NF-κB activities.

### Male Diseases and Female Menopause Syndrome

In TCM point of view, the functions of the reproductive system (including the gonads and reproductive organs) are regulated by the kidney, and the deficiency of kidney essence can cause spermatorrhea, impotence, irregular menstruation, and infertility. (Jeng et al., 1997) found that some fractions of the aqueous extract of *C. officinalis* could enhance the motility of human sperm *in vitro*. The study of (Kam et al., 2012) indicated that *C. officinalis* extract could improve erectile dysfunction (ED). They investigated the relaxation effect of *C. officinalis* extract on the rabbit corpus cavernosum (CC) by an organ bath *in vitro*, and observed the effects on intracavernous pressure (ICP) and cyclic adenosine monophosphate (cAMP) in rats CC tissues after oral administration of the extract for a month. It was found that *C. officinalis* extract showed concentration-dependent relaxation effects on the CC *in vitro*, and significantly elevated the ICP and cAMP concentrations *in vivo*. (Jin et al., 2019) found that the combination of *C. officinalis* and *Psoralea corylifolia* inhibited testosterone-induced benign prostatic hyperplasia (BPH) in rats.

(Chen et al., 2016) found that the iridoid glycosides of *C. officinalis* markedly protected against DM-induced testicular damage, increased the rate of live sperms, and upregulated the levels of testosterone, luteinizing hormone (LH), follicle stimulating hormone (FSH), and gonadotropin-releasing hormone (GnRH). Their study also showed that the iridoid glycosides of *C. officinalis* also had a considerable anti-oxidative and anti-apoptotic effects, which down-regulated the expressions of ROS and MDA, restored the activities of SOD and CAT, and decreased spermatogenic cell apoptosis and Bax/Bcl-2 ratio by inhibiting the activation of the AGEs/RAGE/p38 MAPK pathway.

In addition, *C. officinalis* extract has also been demonstratedto inhibit lipid accumulation in adipocytes, promote osteoblastic differentiation, and increase estradiol production and estrogen receptor *α* (ER*α*) mRNA expression in granulosa cells, and is used to treat obesity and osteoporosis caused by women’s menopausal symptoms (Park et al., 2020a). The above results revealed the therapeutic effect of *C. officinalis* extract on reproductive system diseases at the functional level.

### Cardiovascular Diseases

The treatment of cardiovascular diseases is not a traditional usage of *C. officinalis*, but some experimental studies revealed the cardiovascular protective effects of *C. officinalis*. (Qi et al., 2008) found that the total triterpene acids of *C. officinalis* could alleviate diabetic cardiomyopathy by suppressing the ET/ROS pathway. The results showed that the total triterpene acids of *C. officinalis* alleviated oxidative stress via down-regulating ET-1 expression. It elevated the levels of calstabin, sarcoplasmic reticulum Ca^2+^ ATPase 2a (SERCA2a), and phospholamban (PLB), and improved cardiac function. The research of (Fang et al., 2012) indicated that *C. officinalis* extract could alleviate cardiac hypertrophy. They induced vascular hypertension and cardiac hypertrophy in rats using the “two-kidney two-clip” (2K2C) method. The rats were given intragastric administration of *C. officinalis* extract from the fourth week after the surgery for 4 weeks. The results showed that *C. officinalis* extract reduced the increase in blood pressure caused by 2K2C, reversed the cardiomyocyte hypertrophy and myocardial fibrosis, and down-regulated the expression of p47^*phox*^ and NOX4 in the left ventricular.

Abnormal mitochondrial biogenesis of cardiomyocytes can lead to cardiac dysfunction. (Chen et al., 2015) found that total glycosides and polysaccharides of *C. officinalis* could improve the cardiac function in rats with acute myocardial infarction (AMI). they reduced the area of myocardial infarction, and promoted the mitochondrial biogenesis of cardiomyocytes. Both total glycosides and polysaccharides of *C. officinalis* could increase the mRNA expressions of mitochondrial biogenesis-related genes in myocardial tissue, including genes encoding the subunits of peroxisome proliferators-activated receptor-γ coactivator (PGC)-1*α*, PGC-1*β* and nuclear respiratory factor (NRF-1). The polysaccharides of *C. officinalis* could also reduce glycogen synthase kinase-3*β* (GSK-3*β*) mRNA expression showing a mitochondrial protection effect.

### Antiproliferative and Anticancer Activity

Anticancer treatment is not a traditional usage of *C. officinalis*. But in recent years, some experimental evidences have confirmed the anticancer effect of *C. officinalis*. The research of (Chang et al., 2004) indicated that the aqueous extract of *C. officinalis* could inhibit the proliferation of hepatocellular carcinoma cells (HCC) and significantly suppress the activities of free radicals, xanthine oxidase (XO), and lipid peroxidation *in vitro*.

The combination of selective ER modulators and chemotherapeutic agents is the conventional therapeutic intervention for ER-positive (ER+) breast cancer. The research of (Telang et al., 2012) showed that the aqueous extract of *C. officinalis* could inhibit the growth of ER+ breast cancer. They found that *C. officinalis* extract could inhibit 17*β*-estradiol (E2) -stimulated growth and anchorage-independent colony formation, which indicated an effective reduction of the carcinogenic risk via modulating C2-hydroxylation pathway of E2 metabolism and up-regulating the formation of anti-proliferative metabolite 2-OHE_1_. Triple-negative breast cancer (TNBC) lacks the expression of ER*α*, progesterone receptor, and human epidermal growth factor receptor-2; and is resistant to conventional endocrine or HER-2 targeted therapy. (Telang et al., 2019) found in subsequent research that *C. officinalis* extract could induce cytostatic growth arrest in MDA-MB-231 cells, an *in vitro* model of TNBC, by inhibiting the G1 to S phase transition and decreasing the expression of cyclin D1 and phosphorylated-retinoblastoma proteins. Additionally, *C. officinalis* could promote the apoptosis of tumor cells by up-regulating pro-apoptotic caspase-3/7 activities.

### Osteoporosis

In TCM point of view, the kidney dominates the formation of bone and marrow. The deficiency of the kidney essence can cause diseases of the bones and joints, such as osteoporosis and arthritis. The balance between osteoblasts and osteoclasts helps to maintains the stability of bone mass (Chen et al., 2018). The changes in hormone levels or the condition of inflammation can cause imbalance in bone homeostasis, and lead to osteoporosis and fracture. (Kim et al., 2012) isolated the bone marrow-derived macrophages (BMMs) from the long bones of male ICR mice, and they found that *C. officinalis* extract could decrease the mRNA expressions of the osteoclast-specific gene tartrate-resistant acid phosphatase (TRAP) and osteoclast-associated receptor (OSCAR), and inhibit the differentiation of BMMs to osteoclasts. The results of their research showed that *C. officinalis* extract down-regulated the expressions of c-Fos and nuclear factor of activated T cells cytoplasmic 1 (NFATc1), and suppressed the activation of the upstream p38 mitogen-activated protein kinase (p38MAPK), and c-JUN N-terminal kinase (c-JNK) signaling pathways.

The research of (Park et al., 2020b) showed that the mixture of *C. officinalis* and *Achyranthes japonica* (Niuxi) significantly promoted osteoblast differentiation of MC3T3-E1 mouse preosteoblasts via up-regulating the expressions of osteoblastic differentiation-associated genes, such as alkaline phosphatase (*Alpl*), runt-related transcription factor 2 (*Runx2*), and bone gamma-carboxyglutamic acid-containing protein (*Bglap*). And on the other hand, the mixed extract also inhibited the differentiation of osteoclasts isolated from primary-cultured mouse monocytes. The above research indicated that *C. officinalis* has the ability to regulate osteoblasts and osteoclasts at the same time, which reflected the characteristic of two-ways regulation and homeostasis maintenance of TCM.2.10 Other diseases.

Since the anti-oxidative, anti-apoptotic, and other effects of *C. officinalis* extract have been confirmed (Hwang et al., 2016), some researchers have studied the therapeutic effects of *C. officinalis* in other diseases based on these functional characteristics in recent years. The skin is one of the largest organs of the human body and the most exposed to outdoor contaminants such as particulate matter <2.5 µm (PM_2.5_). (Fernando et al., 2020) found that PM_2.5_ induced ROS accumulation and oxidative stress, and eventually caused cellular apoptosis of human keratinocytes; while the ethanol extract of *C. officinalis* could down-regulate the excessively accumulated Ca^2+^ in the mitochondria of keratinocytes via anti-oxidative effect and protect the skin against PM_2.5_-induced damage.

*C. officinalis* extract has been demonstrated to have an immunomodulatory effect. (Quah et al., 2020) found that the ethanol extract of *C. officinalis* could suppress the IgE-induced degranulation of rat basophilic leukemia cells(RBL-2H3) *in vitro* and had a potential alleviatory effect on allergic dermatitis. The results of their research also showed that loganin, cornuside, and naringenin 7-O-*β*-D-glucoside isolated from *C. officinalis* could potentially disrupt the binding of IgE to human high-affinity IgE receptors (FceRI) by molecular docking analysis. (Du et al., 2008) found that polysaccharides extracted from *C. officinalis* could notably improve the proliferation and transformation of spleen lymphocytes in cyclophosphamide-induced immunosuppressed mice and enhance non-specific immunity, specific humoral immunity, as well as specific cellular immunity, indicating that *C. officinalis* could be used as an adjuvant therapy to patients receiving immunosuppressive treatment.

## Pharmacological Effects of *C. officinalis* Active Components

The active components with specific pharmacological effects are the medicinal material basis for TCM therapeutic effects. The isolation and identification of the active components is conducive to understanding the mechanism of the TCM. The pharmacological effects of more than 20 phytochemicals isolated from *C. officinalis* have been confirmed through experimental studies. We summarized their pharmacological effects in Table 1. Many of the pharmacological effects of these phytochemicals are consistent with those of the *C. officinalis* extract, such as anti-oxidation, anti-apoptosis, anti-inflammatory, anti-cholinesterase, anti-diabetes, and vasorelaxant activity. In addition, some unique effects are found, such as the mucin secretion inhibitory effect of ursolic acid and oleanolic acid, and the antiplatelet aggregation effect of malic acid, succinic acid, and citric acid.

**TABLE 1:** Bioactivities of the main components extracted from *C. officinalis*.

Components	Bioactivities	Diseases/Tissues	Targets (In vitro and In vivo)	Effects	References
Morroniside	Anti- inflammation	Osteoarthritis	In vitro: IL-1β treated primary mouse chondrocytes; in vivo: destabilization of the medial meniscus-treated C57BL/6J mice.	Inhibit NF-κB signaling and proinflammatory NLRP3 expression; down-regulate the expressions of MMP-13 and caspase-1; promote collagen type II and cartilage matrix synthesis.	(Yu et al., 2021)
		Colitis	In vitro: LPS treated colorectal cancer cell; in vivo: DSS induced acute colitis mouse model.	Increase the expression of tight junction proteins; decrease the expressions of pro-inflammatory cytokines; suppress the phosphorylation of STAT3 and NF-κB.	(Yuan et al., 2020)
	Anti-oxidation	Neuropathic pain	In vitro: H_2_O_2_ treated microglial N9 cells, HEK293 cells and HEK293T cells; in vivo: neuropathic pain rat model (L5, L6 spinal nerves ligation).	Active GLP-1 receptors.	(Xu et al., 2017)
	Anti-apoptosis	Diabetic cardiomyopathy	In vitro: primary cultured rat cardiomyocytes.	Down-regulate the expressions of ROS, caspase-3 and bax; up-regulate the expression of bcl-2.	(Pi et al., 2017)
	Anti-diabetes	Diabetic osteoporosis	In vitro: primary cultured rat BMSCs; in vivo: DM rats model (single intraperitoneal injection of 60 mg/kg STZ, SD rats).	Promote osteogenic differentiation of BMSCs; up-regulate the activation and expression of Glo1; down-regulate AGEs formation and RAGE expression.	(Sun et al., 2020)
		Renal tissue	In vitro: AGEs treated mesangial cells.	Inhibit the secretion of ECM major components (LN, FN, and Col-IV) induced by AGEs; inhibit the expressions of RAGE, p38MAPK, NF-κB, and TGF-β induced by AGEs.	(Lv et al., 2016b)
		Liver	In vitro: serum-free DMEM with 50mmol/L glucose treated HepG2 cells; in vivo: DM mice model (single intraperitoneal injection of 60 mg/kg STZ).	Promote glucose uptake; decrease FBG levels in DM mice.	(He et al., 2016)
		Liver	In vivo: db/db mice.	Decrease serum glucose; decrease ROS and lipid peroxidation in liver tissue; down-regulate the expressions of NOX4, P22^phox^, SREBP-1, SREBP-2, Nrf2, HO-1, NF-κB, COX-2, iNOS, MCP-1, bax and Cytochrome C in liver tissue; up-regulate GSH/GSSG ratio.	(Park et al., 2011a; Park et al., 2009)
		Renal tissue	In vivo: db/db mice; STZ (50 mg/kg)-induced DM rats.	Decrease serum TC; decrease renal lipid peroxidation and ROS levels; down-regulate renal SREBP-1, SREBP-2, NF-κB, COX-2 and iNOS expressions; up-regulate GSH/GSSG ratio.	(Park et al., 2010b; Yokozawa et al., 2010)
		Renal tissue	In vivo: STZ (50 mg/kg)-induced DM rats.	Decrease serum glucose, BUN and urinary protein levels; elevate serum albumin and total protein; reduce glycosylated protein and lipid peroxidation; down-regulate RAGE, HO-1.	(Yokozawa et al., 2008)
		Renal tissue	In vitro: AGEs treated rat mesangial cells.	Inhibit AGE-induced mesangial cells proliferation and cell cycle; down-regulate ROS and MDA; up-regulate SOD and GSH.	(Xu et al., 2006)
1,6-α-glucans	Anti-atherosclerosis	Atherosclerosis	In vitro: oxidized-LDL treated RAW264.7 macrophages; in vivo: ApoE^−/−^ mice fed with HFD.	Reduce ox-LDL induced cholesterol levels and inhibit the foam cell formation in RAW264.7 cells; reduce aortic atherosclerotic lesion area in ApoE^−/−^ mice fed with HFD; decrease serum LDL, TC levels, MDA activity, and CD36, SR-A1 expressions; increase SOD activity.	(Zhang et al., 2020)
Loganin	Anti- inflammation	Colitis	In vitro: LPS treated colorectal cancer cell; in vivo: DSS-induced acute colitis mouse model.	Increase the expression of tight junction proteins; decrease the expressions of pro-inflammatory cytokines; suppress the phosphorylation of STAT3 and NF-κB.	(Yuan et al., 2020)
		Ulcerative colitis	In vivo: DSS-induced ulcerative colitis mouse model.	Down-regulate the expressions of IL-6, TNF-α, IL-1β, MCP-1, CXCL10, COX-2 and Sirt1; inhibit the acetylation of NF-κB; reduce macrophage M1 polarization.	(Liu et al., 2020b)
	Anti-diabetes	Renal tissue	In vitro: AGEs treated podocytes; in vivo: KK-Ay mice.	Decrease FBG, Scr and BUN levels; increase serum insulin level; alleviate podocyte loss and apoptosis; activate RAGE/p38 MAPK/NF-κB and RAGE/NOX4/NF-κB pathways in podocytes.	(Chen et al., 2020)
		Liver	In vitro: serum-free DMEM with 50mmol/L glucose treated HepG2 cells; in vivo: DM mice model (single intraperitoneal injection of 60 mg/kg STZ).	Promote glucose uptake; decrease FBG levels in DM mice; down-regulate MDA level and Aldose reductase activity.	(He et al., 2016)
		DM	In vitro: kinetic and molecular docking studies.	Inhibit aldose reductase activity.	(Lee et al., 2015)
		Renal tissue	In vitro: high glucose (30mmol/L) treated HBZY-1 mesangial cells.	Inhibit the expression of FN and IL-6.	(Ma et al., 2014)
		Renal tissue	In vitro: high glucose (27.5mmolL) treated HK-2 cells; in vivo: DM rats model (single intraperitoneal injection of 45 mg/kg STZ).	Inhibit CTGF expression in vitro and in vivo.	(Jiang et al., 2012)
		Liver	In vivo: db/db mice.	Decrease serum glucose and elevate serum leptin; inhibit ROS and lipid peroxidation in the serum and liver; down-regulate the expressions of NOX4 and p22^phox^; down-regulate the expressions of NF-κB, COX-2, iNOS and MCP-1.	(Park et al., 2011b)
		Hepatic and renal tissue	In vivo: db/db mice.	Decrease serum glucose, TG, LDL/VLDL and increase serum HDL; elevate GSH/GSSG ratio; up-regulate PPARα expression; down-regulate SREBP-1, SREBP-2 expressions; inhibit AGEs formation and RAGE expression in hepatic and renal tissues.	(Yamabe et al., 2010)
		Renal tissue	In vitro: AGEs treated rat mesangial cells.	Inhibit AGEs-induced mesangial cells proliferation and cell cycle; down-regulate ROS and MDA; up-regulate SOD and GSH.	(Xu et al., 2006)
	Anti-cholinesterase	Alzheimer's disease	In vivo: scopolamine (1 mg/kg, s.c.)-induced amnesic mice.	Mitigate scopolamine-induced memory deficits in passive avoidance test and Morris water maze test; inhibit acetylcholinesterase activity in the mouse hippocampus.	(Lee et al., 2009)
Ursolic acid	Anti-diabetes	Liver	In vitro: serum-free DMEM with 50mmol/L glucose treated HepG2 cells; in vivo: DM mice model(single intraperitoneal injection of 60 mg/kg STZ).	Inhibit α-glucosidase activity; promote glucose uptake; decrease FBG level in DM mice; down-regulate MDA and Aldose reductase activity, up-regulate SOD activity.	(He et al., 2016)
	Anti-inflammation	Colitis	In vitro: LPS-treated peritoneal macrophages; in vivo: TNBS-induced colitis mice.	Inhibit phosphorylation of IRAK1, TAK1, IKKβ, and IkappaBalpha; inhibit the activation of NF-κB and MAPKs; inhibit IL-1β, IL-6, TNF-α, COX-2 and iNOS expression as well as PGE2 and NO levels; inhibit LPS bind to TLR4 on immune cells.	(Jang et al., 2014)
	Inhibit mucin secretion	Airway diseases	In vitro: EGF (25 ng/mL) or PMA (10 ng/mL) treated NCI-H292 cells (the human pulmonary mucoepidermoid carcinoma cell line).	Inhibit MUC5AC mucin gene expression and mucin protein production.	(Cho et al., 2011)
	Antioxidation	Inner ear diseases	In vitro: H_2_O_2_-treated HEI-OC1 auditory cells.	Reduce lipid peroxidation; up-regulate the activities of CAT and GPX.	(Yu et al., 2009)
Oleanolic Acid	Anti-proliferation	Benign prostatic hyperplasia	In vitro: Human BPH-1 cells; in vivo: BPH rat model (male rats injected with testosterone propionate).	Decrease prostate weight and prostate epithelial thickness; reduce serum DHT and 5α-reductase mRNA levels; down-regulate protein expressions of bcl-2, bcl-xL and PCNA; down-regulate the cell cycle markers CdK4-cyclin D1 and CdK2-cyclin E.	(Cheon et al., 2020)
	Inhibit mucin secretion	Airway diseases	In vitro: EGF (25 ng/mL) or PMA (10 ng/mL) treated NCI-H292 cells.	Inhibit MUC5AC mucin gene expression and mucin protein production.	(Cho et al., 2011)
	Anti-diabetes	DM	In vivo: intraperitoneal inject into the fasting Wistar rats.	Enhance the release of ACh from nerve terminals, stimulate muscarinic M(3) receptors in the pancreatic β cells and augment the insulin release.	(Hsu et al., 2006)
Cornuside	Anti-cholinesterase	Alzheimer's disease	In vitro experiments.	Inhibit ChEs and BACE1 by interacting with both the catalytic active sites and the peripheral anionic sites.	(Bhakta et al., 2017)
	Vasorelaxant activity		In vitro: isometric vascular tone of phenylephrine-contracted thoracic aortae; cGMP production in HUVECs.	Dilates vascular smooth muscle via endothelium-dependent NO/cGMP signaling.	(Kang et al., 2007)
Polymeric proanthocyanidins	Anti-cholinesterase	Alzheimer's disease	In vitro experiments.	Inhibit ChEs and BACE1 by interacting with both the catalytic active sites and the peripheral anionic sites.	(Bhakta et al., 2017)
	Anti-diabetes	DM	In vivo: male Wistar rats with normal blood glucose.	Inhibit α-glucosidase activity; improve the oral sucrose tolerance, and inhibit the rise in the plasma glucose levels in normal rats.	(Park et al., 2011c)
1,2,3-tri-O-galloyl-beta-D-glucose	Anti-cholinesterase	Alzheimer's disease	In vitro experiments.	Inhibit ChEs and BACE1 by interacting with both the catalytic active sites and the peripheral anionic sites.	(Bhakta et al., 2017)
	Anti-diabetes	DM	In vitro experiments.	Inhibit the formation of AGEs and RLAR, inhibit AGE-BSA cross-linking.	Lee et al. (2011)
1,2,3,6-tetra-O-galloyl-beta-D-glucose	Anti-cholinesterase	Alzheimer's disease	In vitro experiments.	Inhibit ChEs and BACE1 by interacting with both the catalytic active sites and the peripheral anionic sites.	(Bhakta et al., 2017)
	Anti-diabetes	DM	In vitro experiments.	Inhibit the formation of AGEs and RLAR, inhibit AGE-BSA cross-linking; prevent the opacity of lenses.	(Lee et al., 2011)
1,2,6-tri-O-galloyl-beta-D-glucose	Anti-diabetes	Diabetes mellitus(DM)	In vitro experiments.	Inhibit the formation of AGEs and RLAR, inhibit AGE-BSA cross-linking; prevent the opacity of lenses.	(Lee et al., 2011)
1,2,4,6-tetra-O-galloyl-bta-D-glucose	Anti-diabetes	Diabetes mellitus(DM)	In vitro experiments.	Inhibit the formation of AGEs and RLAR, inhibit AGE-BSA cross-linking; prevent the opacity of lenses.	(Lee et al., 2011)
1,2,3,4,6-penta-O-galloyl-beta-Dglucose	Anti-diabetes	Diabetes mellitus(DM)	In vitro experiments.	Inhibit the formation of AGEs and RLAR, inhibit AGE-BSA cross-linking; prevent the opacity of lenses.	(Lee et al., 2011)
Tellimagrandin I	Anti-cholinesterase	Alzheimer's disease	In vitro experiments.	Inhibit ChEs and BACE1 by interacting with both the catalytic active sites and the peripheral anionic sites.	(Bhakta et al., 2017)
Tellimagrandin II	Anti-cholinesterase	Alzheimer's disease	In vitro experiments.	Inhibit ChEs and BACE1 by interacting with both the catalytic active sites and the peripheral anionic sites.	(Bhakta et al., 2017)
	Anti-diabetes	DM	In vitro experiments.	Inhibit the formation of AGEs and RLAR, inhibit AGE-BSA cross-linking; prevent the opacity of lenses.	(Lee et al., 2011)
Isoterchebinc	Anti-cholinesterase	Alzheimer's disease	In vitro experiments.	inhibit ChEs and BACE1 by interacting with both the catalytic active sites and the peripheral anionic sites.	(Bhakta et al., 2017)
7-O-Galloyl-D-sedoheptulose	Anti-diabetes	Hepatic tissue	In vivo: db/db mice.	Decrease serum glucose, leptin, insulin, TNF-α, IL-6, resistin, ALT, AST; reduce hepatic AGEs, RAGE and ROS; reduce hepatic p-ERK1/2, p-cJNK, NF-κB, AP-1, MCP-1, ICAM-1, TNF-α, and IL-6.	(Park et al., 2015b)
		DM	In vitro: kinetic and molecular docking studies.	inhibit aldose reductase activity	(Lee et al., 2015)
		Adipose tissue	In vivo: db/db mice.	Decrease serum glucose, leptin, insulin, C-peptide, resistin, TNF-α, IL-6, TG, TC, HDL, VLDL/LDL, ROS and TBARS; up-regulate serum adiponectin; decrease adipose tissue lipid, ROS, TBARS contents and SREBP-1, NF-κB, COX-2, iNOS, MCP-1, ICAM-1, p-cJNK, AP-1, TGF-β1, bax, cytochrome c , caspase-3 expressions; elevate PPARα, PPARγ, and β-cell lymphoma 2 in the adipose tissue.	(Park et al., 2013)
		Renal tissue	In vivo: db/db mice.	Decrease serum insulin, Cr and BUN levels; down-regulate serum TNF-α, IL-6 and ROS; down-regulate renal ROS, TBARS, NF-κBp65, NOX4, p22^*phox*^, COX-2, iNOS, bax and Cytochrome C; up-regulate GSH/GSSG ratio.	(Park et al., 2012)
		Hepatic and renal tissue	In vivo: db/db mice.	Down-regulated the expression of SREBP-1; inhibit AGEs formation and RAGE expression in hepatic and renal tissues.	(Park et al., 2010a)
Malic acid	Anti-platelet		In vitro: platelet viability assay and platelet adhesion assay.	Inhibit platelet aggregation and prevent platelet adhesion.	(Zhang et al., 2013)
Succinic acid	Anti-platelet		In vitro: platelet viability assay and platelet adhesion assay.	Inhibit platelet aggregation and prevent platelet adhesion.	(Zhang et al., 2013)
Citric acid	Anti-platelet		In vitro: platelet viability assay and platelet adhesion assay.	Inhibit platelet aggregation and prevent platelet adhesion.	(Zhang et al., 2013)
7-O-butylmorroniside	Neuroprotection	Neurodegenerative diseases	In vitro: glutamate-treated HT22 hippocampal cells.	Elevate the cell viability (MTT) of glutamate-treated HT22 hippocampal cells.	(Jeong et al., 2012)
5-hydroxymethylfurfuralc (processed *C. officinalis*)	Anti-diabetes	Vascular system	In vitro: high glucos treated HUVECs.	Inhibit HUVECs apoptosis induced by high glucose; reduce ROS and superoxide; down-regulate the expressions of IL-8, JNK1 and JNK2/3; up-regulated the expression of p-Akt.	(Cao et al., 2013)
	Antioxidation	Acute liver injury	In vitro: H_2_O_2_-treated human vein epidermal cell ; in vivo: CCL_4_-induced acute liver injury mice.	Protect human vein epidermal cell against H2O2; decrease ALT, AST in acute liver injury in mice.	(Ding et al., 2008)

Notes: Abbreviations of table 1.

NLRP3: NOD-like receptors three; MMP: matrix metalloprotein; STAT: signal transducer and activator of transcription; GLP: glucagon-like peptide-1; BMSCs: bone marrow mesenchymal stem cells; Glo1: glyoxalase-1; FBG: fasting blood glucose; SREBP: sterol-regulatory element binding proteins; GSSG: oxidized glutathione; HFD: high-fat diet; SR-A1: type A1 scavenger receptor; LPS: lipopolysaccharide; DSS: dextran sodium sulfate; TNF-*α*: tumor necrosis factor-*α*; CXCL10: CXC chemokine ligand-10; CTGF: connective tissue growth factor; TNBS: 2,4,6-trinitrobenzenesulfonic acid; IRAK: interleukin one receptor-associated kinase; IKK*β*: inhibitor of nuclear factor kappaB kinase subunit β; PGE2: prostaglandin E2; TLR4: toll-like receptor four; EGF: epidermal growth factor; PMA: phorbol 12-myristate 13-acetate; GPX: glutathione peroxidase; DHT: dihydrotestosterone; PCNA: proliferating cell nuclear antigen; HUVECs: human umbilical vein endothelial cells; RLAR: rat lens aldose reductase; AP-1: activator protein-1; TBARS: thiobarbituric acid-reactive substance.

## Conclusion

Previously, (Czerwinska and Melzig, 2018) have compared the similarities and differences in main phytochemical components and corresponding biological effects of two edible plants of the same genus, *Cornus mas L.* and *C. officinalis*. The value of their review is to point out that a variety of iridoid glycosides, including morroniside, logmalicids, cornusfurosides, and cornusides, are unique to *C. officinalis* and are closely related to the corresponding biological activities. In their opinion, the phytochemical composition is the important factor determining the biological activities and justifying the traditional usages. They have distinguished the two closely related species, and emphasized the anti-osteoporotic, immunomodulatory effects of *C. officinalis*. However, the medicinal value of *C. officinalis* has not been comprehensively summarized, nor has it been combined with the TCM theories.

According to an authoritative medical book of Ancient China, “Compendium of Materia Medica (Ben Cao Gang Mu)”, C. officinalis “warms and nourishes the liver and kidney, eliminates all diseases caused by “pathogenic wind”, stops menorrhagia, and treats frequent urination in the elderly” (Li, 1975), indicating the therapeutic effects of *C. officinalis* on consumptive disease, strokes and other “wind syndromes” in concept of TCM, gynecological diseases and urinary system diseases. The therapeutic effects of *C. officinalis* on a numbers of diseases have been confirmed by experimental research, but there are still a lot of traditional usages that have not been considered by the modern medical research, such as the treatment of tinnitus and deafness, forgetfulness, hair loss, etc.. At the same time, modern medical research has also found some pharmacological effects beyond the traditional usages of *C. officinalis*, such as protective effects on cardiovascular system and antiproliferative effects on tumors. These studies have deepened the understanding of the pharmacological effects and expanded the indications of *C. officinalis*.

The active components are the material basis of the pharmacological effects of *C. officinalis*. Some of the pharmacological effects of these active components are consistent with those of the *C. officinalis* extract, including anti-oxidation, anti-apoptosis, anti-inflammatory, anti-cholinesterase, anti-diabetes, and vasorelaxant activity, and others are unique, such as the mucin secretion inhibitory effect and the antiplatelet aggregative effect. Iridoid glycosides are the main components with medicinal properties of *C. officinalis*, among them, morroniside and loganin are the indicators for herb quality control according to the Pharmacopoeia of P.R. of China (2015) (Huang et al., 2018).

Traditionally, herbal combinations are more widely used than single herbs in TCM treatment. The different herbs in TCM formula are considered to have synergistic effects or to reduce side effects, that is, the characteristic of “Jun Chen Zuo Shi” of TCM formulas. Some researchers have suggested that combination therapy of two or more natural products may enhance multiple mechanisms of action and presents synergistic effects with fewer side effects than single natural product treatment (Park et al., 2020b). There have been some studies exploring the pharmacological effects and the corresponding mechanisms of herb pairs containing *C. officinalis*. For example, studies showed that the mixed extract of *C. officinalis* and *Morus alba* root and bark could alleviate Osteoarthritis (OA) symptoms of arthroedema, pain and limping, and suppress the expressions of inflammatory markers TNF-α and MMP-13 in the articular cartilage (Park et al., 2020c); the mixture of *C. officinalis* and *Ribes fasciculatum* could ameliorate HFD-induced obesity in C57BL/6J mice, reduce the body fat, decrease the fatty deposition in hepatocytes, and suppress the expressions of adipogenesis-associated genes CCAAT/Cebpa, Fabp4, Pparg, and Srebp1 compared with the model mice (Park et al., 2020d). However, these studies only confirmed that the combination of different herbal medicines could enhance the original pharmacological effects, but did not reflect the synergistic mechanism or the side effects antagonistic mechanism between them. Therefore, new detection technologies are still needed to explain the characteristics of multi-target effects of TCM and the interrelationship between different pharmacological mechanisms more comprehensively.

Finally, *C. officinalis* has been so intensively studied that it appears almost as a panacea for many major conditions. Clinical trials are clearly needed to assess which of the reported pharmacological activities have confirmed efficacy and practical application in humans. (Park et al., 2020e) conducted a randomized, double-blind, controlled trial in Korea and 76 obese female participants were enrolled. The results showed that 12 weeks of administration of the mixture of *C. officinalis* and *Ribes fasciculatum* could significantly decrease the body fat of the participants. However, the clinical studies on the pharmacological effects of single herb of *C. officinalis* are still needed.
